# Effectiveness of a Novel Dentifrice Containing Stabilized Chlorine Dioxide, Sarkosyl, and Sodium Fluoride

**DOI:** 10.3390/dj8040122

**Published:** 2020-10-27

**Authors:** Srinivas Rao Mynenivenkatasatya, Howard Wang, William Cooley, Esmeralda Garcia-Smith, Jaiprakash Shewale, James Ratcliff

**Affiliations:** 1 Department of Periodontology, Stony Brook University, Stony Brook, NY 11794, USA; howardwangdds@gmail.com; 2 Cooley Consulting, Inc., Wyoming, OH 45215, USA; billcooley1930@gmail.com; 3 Rowpar Pharmaceuticals, Inc., Scottsdale, AZ 85260, USA; eagsmith730@gmail.com (E.G.-S.); jshewale@rowpar.com (J.S.); jratcliff@rowpar.com (J.R.)

**Keywords:** stabilized chlorine dioxide, sarkosyl, sodium fluoride, enamel remineralization, anticavity, anticaries, remineralization, biofilm, fluoride uptake, polymicrobial biofilm

## Abstract

This in vitro study evaluated the effectiveness of a novel dentifrice containing stabilized chlorine dioxide, sodium lauroyl sarcosinate (sarkosyl), and sodium fluoride in enhancing enamel fluoride uptake, remineralization, pellicle cleaning and inhibiting biofilm regrowth. Remineralization was measured by fluoride uptake and surface microhardness assessment tests. Artificial stains were removed and scored based on pellicle cleaning ratio. Biofilm regrowth was measured by counting colonies on the agar plates. All studies were conducted using bovine teeth specimens. The efficacy of Toothpaste C (CloSYS anticavity toothpaste) was compared with United States Pharmacopoeia Reference Dentifrice, Toothpaste B (discontinued CloSYS anticavity toothpaste formulation) and leading commercial toothpastes. The enamel fluoride uptake and remineralization by Toothpaste C was 96.1% to 303.3% and 38.0% to 102.4% higher than the tested toothpastes, respectively. The mean pellicle cleaning ratio of Toothpaste C was similar to American Dental Association Reference Material. Toothpaste C had a significant reduction in regrowth of the oral polymicrobial biofilm compared to the control. All tested toothpastes contained 0.24% sodium fluoride. Toothpaste C exhibited significantly superior performance towards fluoride uptake and remineralization compared to the tested toothpastes. Therefore, toothpaste ingredients other than sodium fluoride accounted for the enhanced fluoride uptake and remineralization.

## 1. Introduction

Dental caries is one of the most common preventable chronic diseases affecting human beings [1]. Aside from proper tooth brushing techniques, the selection of a dentifrice is one of the most crucial components for maintaining a healthy dentition and preventing dental caries [2,3]. Akande et al. showed that the use of different brands of dentifrices can lead to varying incidences of caries in an animal model, despite the fact that the dentifrices may all contain fluoride [4]. Guggenheim et al. reported that fluoride dentifrices contribute to a decrease in plaque and caries. Moreover, the performance exhibited by a given dentifrice cannot be explained by the fluoride content alone, but rather by the synergistic effect of the different ingredients in a dentifrice formula [5]. Based on the previously mentioned studies, it is inferred that all fluoridated dentifrices are not equally effective. Evaluation of the effectiveness of a dentifrice in preventing caries and plaque should be based on several objectives: (a) the ability of the dentifrice to maximize the uptake of fluoride for remineralization of the enamel; (b) its ability to adequately clean the teeth; and (c) its ability to inhibit the regrowth of the oral polymicrobial biofilm after brushing [6].

The principal reason for using a fluoridated dentifrice is for the uptake of fluoride onto the enamel surface for remineralization. Several different strategies for enhancing enamel remineralization have been investigated. Studies have demonstrated that: (a) increasing the duration of brushing and (b) increasing the quantity of dentifrice on the toothbrush from 0.5 g to 1.5 g can significantly increase enamel fluoride uptake and the net acid resistance of the dentin post-brushing [7,8]. Other studies have investigated the merits of using stannous fluoride instead of sodium fluoride, showing that a stabilized stannous fluoride (SnF_2_) formula was significantly better at preventing enamel erosion and increasing fluoride uptake than sodium fluoride [9,10]. However, the clinical use of stannous fluoride may have untoward side-effects as it may lead to significantly more staining of the dentition than sodium fluoride or other formulas [11,12]. Other investigators have found initial success in employing the use of novel surfactants, such as sodium dodecyl sulfate and cocaminopropyl betaine, and the use of different remineralization agents such as casein phosphopeptide–amorphous calcium phosphate complexes in preventing enamel demineralization [13,14].

The acquired pellicle is a proteinaceous layer that is almost immediately formed on the enamel surface by the selective adsorption of salivary glycoproteins and acts to protect the tooth from erosive acidic insults [15]. However, the pellicle is also crucial in enabling extrinsic staining of the tooth as the pigmentation is either deposited onto the enamel surface or incorporated into the actual pellicle. The removal of the pellicle eliminates extrinsic stains that otherwise may tenaciously adhere to the enamel surface or imbed within the pellicle itself [16].

A dentifrice may be able to delay the repopulation of the oral polymicrobial biofilm after brushing. Arweiler et al. tested Colgate Total and Parodontax toothpastes against a negative control (water) and a positive control (0.1% chlorhexidine rinse), showing that Colgate Total, Parodontax and 0.1% chlorhexidine rinse were able to reduce biofilm viability significantly (28–50%, 18–31% and 19–50%, respectively) over a period of 24 h compared to using water only [17]. They hypothesized that the effects of the dentifrice are attributable to the residual fluoride and other key ingredients of the toothpaste that persist in the residual plaque, which act as a reservoir. This hypothesis was further substantiated in a study that compared Colgate Total and Crest Pro-Health toothpastes, which showed decreased viability of the biofilm up to 12 h after brushing with these two dentifrices when compared to the control group [18].

The aim of this study is to assess the ability of a novel dentifrice (Toothpaste C) containing stabilized chlorine dioxide, sodium fluoride and sodium lauroyl sarcosinate (sarkosyl) to achieve the aforementioned three goals: (a) to allow for the enhanced uptake of fluoride and remineralization of the enamel; (b) to adequately clean the pellicle; and (c) to inhibit regrowth of the oral polymicrobial biofilm after brushing using proven in vitro laboratory testing techniques.

## 2. Materials and Methods

### 2.1. Toothpaste Samples

Toothpastes B and C are CloSYS Anticavity toothpastes (Rowpar Pharmaceuticals, Inc., Scottsdale, AZ). Toothpaste B’s formulation was improved by replacing sorbitol with sodium lauroyl sarcosinate to generate Toothpaste C, with the aim of achieving better efficacy. Toothpaste B was replaced in November 2017 with Toothpaste C as it had significantly higher efficacy. United States Pharmacopoeia (USP) Reference Dentifrice (Rockville, MD) and American Dental Association (ADA) reference material (Odontex Inc., Lawrence, Kansas, USA) are industry accepted standard reference materials and their composition is not provided by respective suppliers. The ingredients of the tested commercial toothpastes are provided in the Supplementary Materials.

### 2.2. Enamel Fluoride Uptake and Remineralization

Toothpaste B and Toothpaste C were compared to the USP Reference Dentifrice for fluoride toothpaste, Crest 3D White Mild Mint toothpaste and Colgate Total Advanced Whitening toothpaste in their ability to promote enamel fluoride uptake and to promote lesion remineralization under dynamic conditions simulating in vivo caries formation as described in the literature by White [19,20].

Specimen Preparation: In this in vitro study, enamel specimens (n = 18 per treatment group) were isolated from previously extracted bovine teeth. The specimens were cut to 3 mm diameter samples, and their surfaces were further prepared to achieve a high luster polish using gamma alumina. At 200 gF, utilizing a Vickers Diamond indenter, a Vickers hardness number (VHN) value of 25 VHN has an area of approximately 0.015 mm^2^. The area of the 3 mm specimen is 7.069 mm^2^. Therefore, the 3 mm diameter specimens provide a more than adequate surface area to conduct numerous surface microhardness indentions on a single specimen. Also, numerous microhardness indentions can be made in close proximity to one another on a single specimen. Incipient enamel lesions were formed artificially in specimens by exposing the specimens to an acid challenge solution consisting of 0.1 M lactic acid and 0.2% Carbopol C907 that was adjusted to a pH of 5.0 and which was 50% saturated with hydroxyapatite. Each specimen was immersed in the acid challenge solution for 33 h. Following lesion formation, the lesion surface hardness and average lesion depth were measured. The lesion surface hardness of the specimens ranged from 25–45 according to the Vickers microhardness test (VHN; 200 gF, 15 s dwell time) and the average lesion depth was approximately 70 µm. Cross-sectional microhardness was utilized to determine the average lesion depth.

Procedure: Each enamel specimen was placed in the lesion-forming solution for 4 h per day. Following exposure to the lesion-forming solution, each sample received 4 one-minute dentifrice treatments. After each dentifrice treatment, the specimens were rinsed with distilled water. The specimens were then placed in human saliva for the remaining time (~20 h). This regimen was repeated for 10 days and interim surface microhardness (SMH) measurements were obtained. The specimens were then subjected to an additional 10 days of the treatment regimen for a total of 20 days. SMH assessments were conducted after 10 and 20 days.

Analysis: The fluoride content of each specimen was analyzed at the end of the treatment regimen (after 20 days). Samples were taken from each enamel specimen using a micro-drill technique to a depth of 100 µ. The diameter of the drill hole was determined microscopically. The enamel powder from the drill hole was collected, dissolved (20 µL of HClO_4_, 40 µL citrate/EDTA buffer and 40 µL deionized water) and analyzed with a fluoride ion selective electrode (ISE). The mV readings obtained were compared to a similarly prepared fluoride standard curve for fluoride concentration determination. Fluoride content was calculated in µg F/cm^3^ (µg F × dilution factor ÷ volume of drilling), as described by White [19,20]. In addition to evaluating fluoride uptake, lesion surface hardness of the enamel specimens was also analyzed at baseline, 10 days and 20 days. The differences in lesion surface hardness observed demonstrated the ability of the dentifrice treatment to enhance remineralization of the enamel surface.

Statistical Analysis: Statistical analyses were performed with a one-way analysis of variance model using Sigma Plot Software (13.0). Individual mean differences were analyzed by the Student–Newman–Keuls (SNK) test.

### 2.3. Pellicle Cleaning

Toothpaste B and Toothpaste C were studied for their cleaning ability to remove stained pellicle. The control dentifrice was ADA reference material. The methodologies were adopted from Stookey et al. [21].

Specimen Preparation: The enamel specimens were isolated from previously extracted bovine teeth. The specimens were cut into 10 × 10 mm samples, placed in an autopolymerizing methacrylate resin, smoothed and polished. They were also lightly etched to allow for increased stain accumulation and adherence. They were then positioned on rotating rods and placed in a 37 °C incubator. The specimens were then alternately exposed to air or staining broth consisting of peptone–glucose–yeast extract (PGY) broth, tea, coffee, mucin, FeCl_3_, and *Micrococcus luteus* to develop stains; the procedures used followed a previously developed model for enamel pellicle creation and staining [21]. The specimens were rinsed and replenished with staining broth daily for 7 days for uniform stain accumulation. Following application of the staining broth, all specimens were rinsed, air dried, and refrigerated until future use. The grade of stain on the enamel specimens was standardized photometrically so that all treatment groups had the same average baseline score of stain. Using a spectrophotometer (Minolta CM2600d), a quarter-inch diameter circle in the center of each sample was graded photometrically according to the L value of the L*a*b* scale. Specimens graded between 30 and 42 (30 = more darkly stained) were selected. On the basis of these scores, the specimens were divided into groups of 80 specimens each, with each group having approximately the same average baseline score. The specimens were then processed for the pellicle cleaning protocol. Specimens were distributed into the test groups following a stratified randomization procedure. This eliminated any bias effect.

Procedure: The stained enamel specimens were installed on a mechanical V-8 cross-brushing machine outfitted with soft, nylon-filament toothbrushes (Oral-B 40-Soft Toothbrushes). The surface tension was adjusted to 150 g. The dentifrices were prepared as slurries (25 g of dentifrice in 40 mL of deionized water. Each enamel specimen was brushed with dentifrice slurry for 800 strokes (4.5 min); fresh dentifrice slurry was made after brushing four specimens. Following the brushing cycle, enamel specimens were rinsed, blotted dry, and scored for staining photometrically using the L value of the L*a*b* scale.

Statistics: The mean decrement between the pre- and post-brushing stain scores was determined for the control group and assigned a pellicle cleaning ratio (PCR) value of 100. A constant value was calculated by dividing the mean decrement of the ADA Control into 100. The individual PCR value for each specimen was calculated by multiplying its individual decrement by the calculated constant. The mean, standard deviation, and standard error of the mean (SEM) for each test group were then calculated using the individual PCR values. The larger the PCR value, the greater the amount of stained pellicle removed from the enamel surface in this test. Data were analyzed using a one-way analysis of variance model (IBM SPSS Statistics 24 Software). Data were further analyzed using pairwise multiple comparison procedures (Student–Newman–Keuls method). All analyses were performed with the significance level set at 0.05.

### 2.4. Inhibition of the Regrowth of Polymicrobial Biofilm

Toothpaste C was subjected to additional testing to assess its effectiveness in the inhibition of the regrowth of the oral polymicrobial biofilm. Pooled saliva from multiple donors was grown over night in brain heart infusion (BHI) broth supplemented with yeast extract (YE), vitamin K and hemin (BHI-YE) to generate a mixed species whole salivary bacterial preparation. Each enamel specimen was sterilized with ethylene oxide. BHI-YE (3 mL) was inoculated with 50 µL of an overnight culture of the mixed species whole salivary bacterial preparation in the wells of a tissue culture plate containing sterilized 4 × 4 mm bovine enamel sections (embedded in 12 × 12 × 7 mm acrylic resin and 1 section/well). The plates were incubated for 24 h to grow the biofilm on the enamel. To remove the biofilm, the bovine enamel sections were brushed individually with Toothpaste C (3 sections/paste) for 30 s (similar time interval to brushing by human subjects). Brushing with water served as a control. The enamel specimens were brushed with an automated brushing machine. The toothbrushes were all new brushes (freshly opened) and, therefore, were not contaminated. Toothpaste C met standard microbial tests for aerobic plate count, fungi and yeast, and presence of pathogens. Each enamel specimen containing the biofilm and its brush was contained in a sealed sterile glass cylinder and so there was no chance for cross contamination. Furthermore, the use of a whole salivary mixed species model used in this study minimizes any possible contamination because of the huge number of organisms and large number of species. Therefore, all measures were taken to avoid any cross contamination. After brushing, the bovine enamel sections were rinsed with sterile water and inserted into a fresh tissue culture plate containing 3 mL of BHI-YE to facilitate regrowth of the remaining oral biofilm on the enamel sections. The plates were incubated for 24 h. The bovine enamel sections were removed, placed in 2 mL of sterile saline, sonicated for 10 sec, vortexed for 10 sec, diluted to 1:10 and 1:1000 and spiral plated on blood agar plates. After 24 h of incubation, the colonies on the agar plates were counted by an automated colony counter using the method described by Sabrah et al. [22].

## 3. Results

### 3.1. Enamel Fluoride Uptake and Remineralization

**Figure 1:**
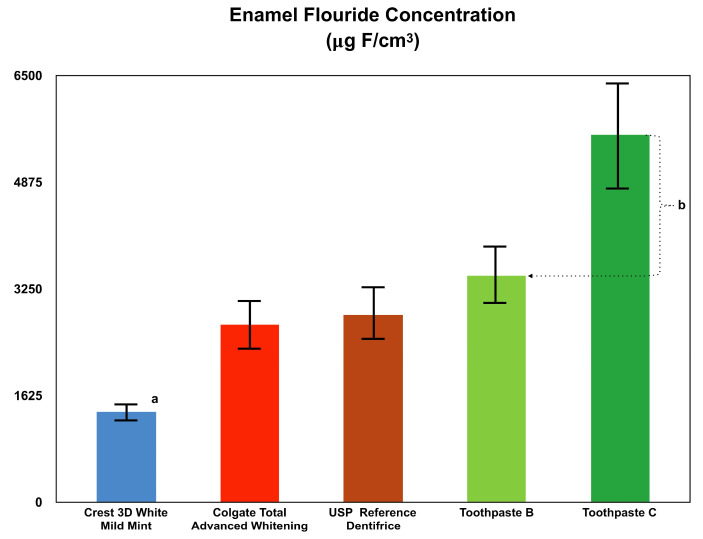
Comparison of enamel fluoride uptake between Crest 3D White Mild Mint, Colgate Total Advanced Whitening, United States Pharmacopoeia (USP) Reference Dentifrice, Toothpaste B, and Toothpaste C. Eighteen replicates (n = 18) of each toothpaste were run and individual mean differences were analyzed by Student–Newman–Keuls analysis. ^a^ Brackets represent two standard deviations, mean ± one standard deviation. ^b^ Significant difference, *p* < 0.001.

Enamel fluoride concentration measured after 20 days is presented in Figure 1. Average fluoride concentration (based on numerous in vitro enamel fluoride uptake studies where the baseline fluoride concentrations were determined) in the single-sourced bovine enamel utilized as the test substrate is less than 100 ppm. Therefore, the gross enamel fluoride concentrations determined after 20 days provide an appropriate comparative representation of the efficacy of each treatment to deliver fluoride into the demineralized enamel substrate i.e., fluoride uptake. Toothpaste C exhibited 62.3% more mean fluoride uptake into incipient lesioned enamel than Toothpaste B. The mean fluoride uptake of Toothpaste C was 96.1%, 107.7%, and 303.3% higher than USP Reference Dentifrice, Colgate Total Advanced Whitening, and Crest 3D White Mild Mint toothpastes, respectively. The Toothpaste C treatment group exhibited significantly greater enamel fluoride uptake compared to all other treatment groups (*p* < 0.001).

**Figure 2:**
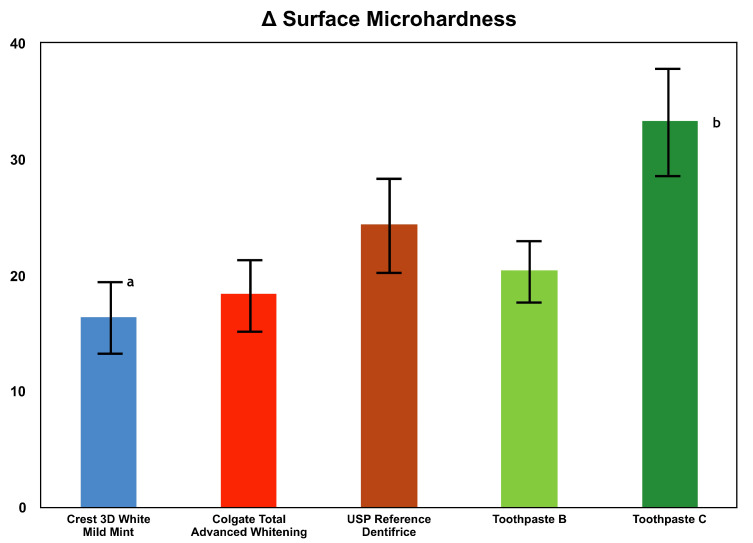
Comparison of Δ surface microhardness after 20 days of remineralization treatment between Crest 3D White Mild Mint, Colgate Total Advanced Whitening, USP Reference Dentifrice, Toothpaste B, and Toothpaste C. ^a^ Brackets represent two standard deviations, mean ± one standard deviation. ^b^ Significant difference, *p* < 0.001, large effect size for Toothpaste C over all others with Cohen’s *d*: 1.64.

The protocol for the remineralization study involved repeated acid challenge and remineralization treatment. Therefore, a net increase in SMH is the combined result of enhanced remineralization and reduced demineralization. Toothpaste C exhibited 69.3% more remineralization (ΔSMH) after 20 days than Toothpaste B (Table 1 and Figure 2). The remineralization (ΔSMH) after 20 days by Toothpaste C was 38.0%, 81.5%, and 102.4% higher than USP Reference Dentifrice, Colgate Total Advanced Whitening, and Crest 3D White Mild Mint toothpastes, respectively. The toothpaste C treatment group exhibited significantly greater surface remineralization compared to the USP Reference and compared to all other treatment groups following 10 days of treatment (*p* = 0.004). Similarly, the Toothpaste C treatment group exhibited significantly greater surface remineralization compared to all other treatment groups following 20 days of treatment (*p* < 0.001).

### 3.2. Stained Pellicle Cleaning

When compared to the ADA reference dentifrice and Toothpaste B, Toothpaste C was the most effective in the removal of stained pellicle (Table 2). Compared to the ADA reference dentifrice, Toothpaste B was less effective at removing stained pellicle. The difference in the removal of stained pellicle by Toothpaste C compared to Toothpaste B and the ADA Reference Material was statistically significant (*p* < 0.05).

### 3.3. Inhibition of the Regrowth of the Polymicrobial Oral Biofilm

Table 3 presents the results of biofilm viability as measured by colony forming units (CFU) per mL (CFU/mL) assay. There was a significant reduction in the regrowth of the oral polymicrobial biofilm obtained by Toothpaste C compared to the water-brushed control. The mean CFU/mL for Toothpaste C was 5.08 × 10^7^ compared to 13.1 × 10^7^ in the control group. Therefore, the results demonstrate that Toothpaste C is highly effective at reducing bacteria in oral polymicrobial biofilms (*p* = 0.018).

## 4. Discussion

Research to develop a multifunctional dentifrice for achieving good oral health is ongoing. The criteria for such a toothpaste should include enhanced enamel remineralization, removal of stained pellicle, and inhibition of biofilm regrowth, which leads to the prevention of both caries and periodontal diseases. Much of this research can only be performed in the laboratory setting due to many other factors that can be introduced in clinical practice, such as different brushing techniques and dietary variations of subjects that will impact the outcome. Nevertheless, Johannsen et al. found a positive correlation between in vitro studies and in vivo findings [23]. Also, the Stookey technique for measuring stain removal used in this study has been validated in numerous prior studies [21,24]. Therefore, it is reasonable to extrapolate the in vitro findings of the present research to clinical settings.

Enamel fluoride uptake and remineralization are major reasons for using a fluoride-containing toothpaste. All tested toothpastes contained about 0.24% sodium fluoride. However, each dentifrice exhibited different levels of enamel fluoride uptake and remineralization, demonstrating the critical role of other ingredients in the efficacy of toothpaste. As evident in the results, Toothpaste C outperformed all other tested dentifrices in both enamel fluoride uptake as well as remineralization. This finding aligns with other studies showing that not all fluoride-containing dentifrices are able to achieve a high level of fluoride uptake and subsequent remineralization [25]. A limitation of this study was that the surface microhardness properties were not confirmed using SEM observation or micro CT imaging.

The different formulations play a large role in enamel fluoride uptake. Previous studies have shown that enamel fluoride uptake is not only influenced by free available fluoride, but more so by other factors [25]. This is demonstrated in the results of this study, where although Toothpaste B and Toothpaste C contain comparable amounts of sodium fluoride and stabilized chlorine dioxide, Toothpaste C incorporated sarkosyl and resulted in statistically more fluoride uptake into incipient lesioned enamel than Toothpaste B. The results from the surface microhardness study also showed the superiority of Toothpaste C to Toothpaste B, as well as all other tested dentifrices. This can be interpreted as a combined result of enhanced remineralization and reduced demineralization due to the efficacy of Toothpaste C in both aspects of enamel fluoride uptake and remineralization.

The synergistic effect of fluoride to certain compounds has been previously investigated. Da Camara et al. found that 1100 ppm of fluoride combined with sodium hexametaphosphate in a conventional toothpaste significantly reduces enamel demineralization in situ when compared to 1100 ppm of fluoride alone [26]. In addition, other studies showed that arginine promotes enamel fluoride uptake into artificial lesions in an in vitro model [27].

Sarkosyl is a surfactant and may allow for a deeper penetration of stabilized chlorine dioxide and sodium fluoride. Chlorine dioxide released from stabilized chlorine dioxide is known to disrupt biofilms [28], and may also enhance the uptake of fluoride on the enamel surface. Toothpastes B and C contain comparable amounts of stabilized chlorine dioxide; however, Toothpaste B does not contain sarkosyl, but does contain the sweetener sorbitol. Eliminating sorbitol in Toothpaste C increased its stability as polyhydroxy alcohols like sorbitol react with stabilized chlorine dioxide, making the formula less stable (US Patent Application No. 13/115,815). The bioavailability of chlorine dioxide in Toothpaste B may be diminished due to the destabilizing effect of sorbitol. Thus, only Toothpaste C was examined for its ability to penetrate oral biofilms. Therefore, another limitation of the current research was that the ability of Toothpaste B to inhibit plaque regrowth was not tested. The specific mechanism of the observed synergism between sodium fluoride, stabilized chlorine dioxide, and sarkosyl remains under investigation. Their combined effects are elucidated in the results of the experiments performed in this study and are the subject of ongoing research.

In the experiment to assess the ability of Toothpaste B, Toothpaste C, and the ADA reference material to clean the pellicle, it was found that Toothpaste C was the most effective, followed by the ADA reference material and Toothpaste B. These differences were found to be statistically significant. Interestingly, the difference in formula between Toothpastes B and C was only the addition of sarkosyl and the elimination of sorbitol. Sarkosyl is an ionic surfactant that functions both as a foaming agent and surfactant. The results presented here show that there is a symbiotic effect of the addition of sarkosyl to fluoride and stabilized chlorine dioxide, as evidenced by the increased efficacy of Toothpaste C compared to Toothpaste B in cleaning the pellicle.

Preventing the regrowth of the biofilm is an important step in achieving good oral health, as biofilm formation is the first step in the development of plaque. The ability of Toothpaste C to inhibit oral biofilm regrowth has been found to be superior to brushing with water alone. This finding has particular significance as prior literature has shown that brushing with dentifrice does not necessarily enhance plaque removal, or has a weak inhibitory effect on biofilm regrowth compared to brushing with water alone [16,29,30]. The removal of the pellicle is essential to prohibit polymicrobial biofilm formation, which may lead to demineralization.

Published studies show the difference between plaque removal with or without a dentifrice. The reason for this difference is controversial. A common hypothesis is that if there is adequate mechanical removal of the biofilm by brushing alone, then the added effect of brushing with a dentifrice is likely to be negligible. However, even with the most excellent oral hygiene regimens, biofilm is always left behind, and the regrowth of the oral polymicrobial biofilm is directly proportional to the quantity of residual bacteria left after brushing [31]. In the study reported here, both the experimental and control groups utilized the same brushing regimen. The results demonstrate that Toothpaste C is highly effective in reducing bacteria in the oral polymicrobial biofilm, as the biofilm viability count was decreased by more than half compared to the control group. Stabilized chlorine dioxide has long been used in a safe and efficacious mouth rinse that can even aid in promoting the healing of medication-related osteonecrosis of the jaw, and its use in dentifrices is an appealing advent in oral healthcare products [32].

## 5. Conclusions

Sodium fluoride, stabilized chlorine dioxide and sarkosyl in the novel dentifrice tested showed a synergistic phenomenon as its effectiveness in the three parameters tested were greater than all other dentifrices studied. Better pellicle removal by Toothpaste C (the current commercial product) may be attributed to increased penetration of chlorine dioxide in presence of sarkosyl, thereby disrupting the biofilm and eliminating the anaerobic bacteria residing in the polymicrobial biofilm. The elimination of anaerobic bacteria by Toothpaste C results in reduced observed regrowth of the biofilm. Disruption of the polymicrobial biofilm further enhances the penetration of sodium fluoride, thereby resulting in much higher fluoride uptake and enhanced remineralization as demonstrated by increased surface microhardness.

## Figures and Tables

**Table 1:** Surface microhardness after 10 and 20 days of remineralization treatment.

Toothpaste	Sodium Fluoride	Surface Microhardness (SMH) *				
		Baseline (Pre-Test) Mean SMH ± SEM	After 10 Days		After 20 Days	
			Mean SMH ± SEM	Δ SMH ± SEM	Mean SMH ± SEM	Δ SMH ± SEM
Crest 3D White Mild Mint	0.243%	32.1 ± 1.1	43.4 ± 2.1	11.3 ± 1.5	48.6 ± 2.2	16.5 ± 1.6
Colgate Total Advanced Whitening	0.24%	32.1 ± 1.1	45.4 ± 1.9	13.3 ± 1.4	50.5 ± 2.1	18.4 ± 1.5
USP Reference Dentifrice	0.24%	32.1 ± 1.1	48.9 ± 2.0	16.8 ± 1.3	56.3 ± 2.7	24.2 ± 2.0
Toothpaste B	0.24%	32.2 ± 1.1	46.5 ± 2.0	14.3 ± 1.2	51.9 ± 2.2	19.7 ± 1.3
Toothpaste C	0.24%	32.2 ± 1.1	54.9 ± 2.1	22.7 ± 1.4	65.6 ± 2.5	33.4 ± 2.2

* Eighteen replicates (n = 18) of each toothpaste were run. Individual mean differences of total surface microhardness were analyzed by Student–Newman–Keuls analysis. SEM = standard error of the mean.

**Table 2:** Pellicle cleaning ratio of dentifrices. ADA = American Dental Association.

Toothpaste	n	Pellicle Cleaning Ratio	
		Mean	SEM
ADA Reference Material	75	100.00	± 1.20
Toothpaste B	76	94.48	± 1.12
Toothpaste C	75	103.51	± 0.96

**Table 3:** Regrowth of polymicrobial oral biofilm. CFU = colony forming units.

Sample Number	Mean CFU/mL	Group Mean CFU/mL	*p* Value Compared to Control
Toothpaste C 1	1.04 × 10^7^	5.08 × 10^7^	0.018
Toothpaste C 2	1.39 × 10^8^		
Toothpaste C 3	3.18 × 10^6^		
Control 1 *	8.31 × 10^7^	13.1 × 10^7^	NA
Control 2	9.96 × 10^7^		
Control 3	2.09 × 10^8^		

* Brushing with water served as a control.

## Supplementary Materials

The following are available online at https://www.mdpi.com/2304-6767/8/4/122/s1, Table S1: Ingredients of tested commercial toothpastes.
