# Supplementary material for: Effectiveness of a Novel Dentifrice Containing Stabilized Chlorine Dioxide, Sarkosyl, and Sodium Fluoride
Source: Dent J (Basel). 2020 Oct 27;8(4):122. doi: 10.3390/dj8040122 (PMC7712167; doi:10.3390/dj8040122)
Supplement: Supplementary file 1 [file dentistry-08-00122-s001.pdf]

dentistry journal

Supplementary Materials: Effectiveness of a Novel Dentifrice Containing Stabilized Chlorine Dioxide, Sarkosyl, and Sodium Fluoride

Srinivas Rao Mynenivenkatasatya *, Howard Wang, William Cooley, Esmeralda Garcia-Smith, Jaiprakash Shewale and James Ratcliff

Table S1. Ingredients of tested commercial toothpastes.

Ingredient	ClōSYS Anticavity		Crest 3D White Mild Mint***	Colgate Total Advanced Whitening****
	Toothpaste B*	Toothpaste C**		
Sodium Fluoride	0.24%	0.24%	0.243%	0.24%
Triclosan	---	---	---	0.30%
Stabilized Chlorine Dioxide	+++	+++	---	---
Peppermint oil blend	+++	+++	---	---
Menthol crystals	+++	+++	---	---
Sucralose	+++	+++	---	---
Disodium hydrogen phosphate	+++	+++	+++	---
Sodium dihydrogen phosphate	+++	+++	---	---
Cellulose gum	+++	+++	---	+++
Titanium dioxide	+++	+++	+++	+++
Hydrated silica	+++	+++	+++	+++
Sodium lauroyl sarcosinate (sarkosyl)	+++	+++	---	---
Sorbitol	+++	---	+++	+++
Flavor (proprietary)	---	---	+++	+++
Sodium Hydroxide	---	---	+++	+++
Sodium Lauryl Sulfate	---	---	+++	+++
Alcohol	---	---	0.7%	---
Xanthan Gum	---	---	+++	---
Sodium Saccharin	---	---	+++	+++
Poloxamer 407	---	---	+++	---
Glycerin	---	---	+++	+++
Carbomer	---	---	+++	---
Polysorbate 80	---	---	+++	---
Sodium Benzoate	---	---	+++	---
Cetylpyridinium Chloride	---	---	+++	---
Benzoic Acid	---	---	+++	---
Copernicia Cerifera (Carnauba) Wax	---	---	+++	---
Blue 1	---	---	+++	---
Yellow 5	---	---	+++	---
PVM/MA copolymer	---	---	---	+++
Propylene glycol	---	---	---	+++
Carrageenan	---	---	---	+++
Water	+++	+++	+++	+++

* US Patent Application Number 13/131,506.

**US Patent Application No. 16/133,359.

***https://www.heb.com/product-detail/crest-3d-white-whitening-toothpaste-mild-mint/2096274; accessed on October 12, 2020.
****https://dailymed.nlm.nih.gov/dailymed/drugInfo.cfm?setid=219a0e97-f89b-4cde-9965-3d25b6896573##; accessed on October 12, 2020.
